# Step Counter Use and Sedentary Time in Adults: A Meta-Analysis

**DOI:** 10.1097/MD.0000000000001412

**Published:** 2015-09-04

**Authors:** Shanhu Qiu, Xue Cai, Changping Ju, Zilin Sun, Han Yin, Martina Zügel, Stephanie Otto, Jürgen M. Steinacker, Uwe Schumann

**Affiliations:** From the Department of Endocrinology (SQ, XC, CJ, ZS, HY), Zhongda Hospital, Institute of Diabetes, School of Medicine, Southeast University, Nanjing, P.R. China; and Department of Internal Medicine II (HY, MZ, SO, JMS, US), Division of Sports and Rehabilitation Medicine, Ulm University, Ulm, Germany.

## Abstract

Although step counters are increasingly being used in walking programmes to promote sedentary behavior changes in adults, their effectiveness remains unknown. The aim of this meta-analysis of randomized controlled trials (RCTs) was to assess the effectiveness of step counter use in reducing sedentary time among adults.

English-language RCTs from 3 databases were searched up to December 2014. Studies were included if they evaluated the effects of step counter use in adult populations and reported outcomes in sedentary time. Summary estimates (Cohen *d* with 95% confidence intervals [CIs]) were pooled using a random-effects model. Subgroup analyses and random-effects meta-regression analyses based on the characteristics of participants or interventions were conducted to explore their associations with sedentary time changes.

Fifteen RCTs with a total sample size of 3262 adults were included. Step counter use was associated with a small but significant overall effect in reducing sedentary time (*d* = −0.20, 95% CI −0.33 to −0.07), equating to a reduction in sedentary time of ∼23 min/d compared with controls. Subgroup analyses showed that step counter use with a step goal was associated with significantly reduced sedentary time (*d* =− 0.32, 95% CI −0.53 to −0.11), whereas without, it had only a trend. A greater reduction in sedentary time was observed among step counter users employing objective methods than those employing subjective methods for measurement (*P* = 0.03). Effects of covariates on sedentary time changes were generally unclear.

Step counter use is associated with reduced sedentary time among adults. Future studies are required to specify the step goal use and to employ objective as well as subjective methods for measuring both total and domain-specific sedentary time.

## Abstract 2

Supplemental Digital Content is available in the text

## INTRODUCTION

Sedentary time, which refers to time spent sitting or reclining during waking hours while having an energy expenditure no >1.5 metabolic equivalents,^1^ has emerged as an independent risk factor for obesity, metabolic syndrome, type 2 diabetes, cardiovascular disease, and cancer in adults.^2–5^ A recent meta-analysis by Biswas et al^6^ has also noted that prolonged sedentary time is independently associated with a greater risk for all-cause, cardiovascular, and cancer mortality, regardless of physical activity. However, more than one half of the waking time in adults is spent being sedentary,^7^ even in those who already meet current physical activity recommendation of ≥150 min/wk of moderate-to-vigorous physical activity sustained in bouts lasting 10 minutes or longer.^8^ These concerns therefore support the notion that the development and evaluation of effective interventions to reduce sedentary time in tandem with the traditional recommendation to increase physical activity in adults is a priority.

In recent years, step counters, such as pedometers and accelerometers, are increasingly being used in walking programmes in community and workplace settings to promote physical activity and to reduce sedentary time among adults.^9–24^ Although it has been well documented that step counter use is associated with increased physical activity,^25,26^ controversy still remains regarding its effectiveness in reducing sedentary time. Several randomized controlled trials (RCTs) pointed out that step counter use could significantly reduce sedentary time in adults;^11,19,23^ however, others argued that there were only minor or no effects.^9,10^ Moreover, previous meta-analyses have shown that having a step goal, such as 10,000 steps/d or an alternative step goal, is key to an increase in physical activity among step counter users.^25,26^ Yet it is not known whether such a step goal use will also lead to a significant reduction in sedentary time, although there is some evidence that increased physical activity is positively associated with decreased sedentary time (that is, physical activity displaces sedentary time).^27^

Therefore, the aims of this meta-analysis of RCTs were to determine the association between step counter use and sedentary time, as well as to assess the importance of step goal setting in reducing sedentary time among adults.

## MATERIALS AND METHODS

### Data Sources and Searches

This study followed the procedures for a meta-analysis as documented in the Preferred Reporting Items for Systematic Reviews and Meta-Analyses (PRISMA) statement (Table, Supplemental Digital Content 1, http://links.lww.com/MD/A390, which shows the PRISMA checklist),^28^ and reported with reference to a prospectively registered protocol in PROSPERO (CRD42015016888; Table, Supplemental Digital Content 2, http://links.lww.com/MD/A390, which describes the registered protocol). Searches were restricted to peer-reviewed English-language research articles in the databases of PubMed, Web of Science, and the Cochrane Central Register of Controlled Trials from inception through December 12, 2014. Search strategies were built around 2 groups of text words or Medical Subject Heading terms related to step counters and sedentary time, along with entry terms associated with a sensitive search filter for RCTs (Table, Supplemental Digital Content 3, http://links.lww.com/MD/A390, which shows the search strategies).^29^ In addition, manual searches of reference lists from relevant publications, systematic reviews, or meta-analyses were conducted to supplement the electronic searches. Gray literature such as dissertations and unpublished data were not sought because it was impractical to identify them from all authors and institutions around the world.

### Study Selection

The criteria for inclusion were defined based on the “PICOS” principle, that is, participants, interventions, comparisons, outcomes, and study design. Studies were included if they included adult populations (mean ages ≥18 years), used step counters (eg, pedometers, accelerometers) as the intervention for physical activity motivation (eg, walking more), compared with control groups that received usual care, were asked to maintain current lifestyle, or received interventions that had nothing to do with physical activity or sedentary behavior, reported change scores or postintervention values of sedentary time (eg, time spent being sedentary or sitting) assessed by subjective (eg, International Physical Activity Questionnaire [IPAQ]) or objective methods (eg, accelerometers), and were RCTs. To assess the long-term effect of step counter use in reducing sedentary time among adults, the length of step counter intervention was restricted to 8 weeks or longer—a time window that is widely used for evaluating the intervention effects on health outcomes or metabolic profiles.^29,30^

Studies were excluded if they included children or adolescents, used step counters only for measuring physical activity or sedentary time, or had step counters sealed in the intervention groups or unsealed in the control groups. Studies were also excluded if they enrolled adults requiring to be hospitalized (eg, inpatients), were posters or nonrandomized studies, or did not report outcomes in sedentary time including the case that such information could not be obtained from the corresponding authors.

### Data Extraction and Quality Assessment

All retrieved citations together with the abstracts were downloaded to EndNote X5 (Thompson Reuters, San Francisco, CA) and duplicates were removed using “duplicate” function or by hand. Following initial title and/or abstract screen, full-text articles were retrieved for any studies deemed appropriate or of uncertainty about their eligibility. Data were extracted from studies that met all inclusion criteria using a standardized data collection form, which included characteristics of study participants (including population sources, number of participants, mean ages, and body mass index [BMI] at entry, sex [proportion of women], and baseline mean sedentary time [unit, min/d]); characteristics of study interventions (including description of the interventions [eg, step goal use, components on sedentary behavior changes, methods for sedentary time measurement] and length of interventions); characteristics of control groups; outcomes of interest (changes in sedentary time or postintervention values [unit, min/d]); details of study sources (including authors and date of publication). In addition, average baseline values and change scores of total walking steps (unit, steps/d) and physical activity (including light, moderate, vigorous, moderate-to-vigorous, and total physical activity [unit, min/d]) were also extracted.

The Cochrane risk of bias tool was used to assess the methodological quality within included RCTs.^31^ For each RCT, 6 domains were judged to be of high, low, or unclear risk for bias: sequence generation, allocation concealment, blinding of participants and personnel, blinding of outcome assessment, incomplete outcome data, and selective outcome reporting. These 6 domains assess the level of risk regarding selection bias, performance bias, detection bias, attrition bias, and reporting bias.

Two independent authors (S.Q. and X.C.) conducted the literature selection, data extraction, and quality assessment in an unblinded manner. When disagreements occurred, consensus was achieved through discussion with a third author (U.S.).

### Data Synthesis and Analysis

Change scores from baseline or postintervention values of sedentary time expressed as means with standard deviations from studies using intention-to-treat or per-protocol analyses were entered in the same meta-analysis.^31^ For studies that did not report means, they were imputed by using medians directly. For studies that did not report standard deviations, they were calculated from standard errors, 95% confidence intervals (CIs), interquartile ranges, or ranges.^31,32^ For studies that had 2-step counter intervention or control groups, these groups were combined into 1 group to create a single pairwise comparison and to overcome a unit-of-analysis error.^31^ Moreover, for studies that reported outcome variables at different time points within the intervention period, outcomes from the last time point were used for primary analyses.^31^

Outcome estimates expressed as Cohen *d* and 95% CIs were assessed using a random-effects model, which better accounts for between-study heterogeneity than a fixed-effects model.^31^ The reason for choosing Cohen *d* as the effect size rather than weighted mean differences was due to the different methods in measuring sedentary time (questionnaires versus instruments). Interpretation of the effect size was based on Cohen criteria, where *d* < 0.40 represents a small effect, 0.40 to 0.70 a medium effect, and >0.70 a large effect.^33^ Statistical heterogeneity was assessed using the Cochrane *Q* test (considered the *Q* test with a *P* < 0.10 as heterogeneous) and the *I*^*2*^ index (considered an *I*^*2*^ value ≥50% as heterogeneous).^31^

Subgroup analyses based on step goal use (with versus without) and components focused on sedentary behavior changes (with versus without) were performed to evaluate their influences on outcome estimates. Another subgroup analyses on the basis of subjective and objective methods were conducted to compare their sensitivities or accuracies in determining sedentary time. Univariate, weighted random-effects meta-regression analyses were carried out to assess whether the changes in outcome estimates could be mediated by the following factors: baseline mean age (logarithmic transformation), BMI, sedentary time and physical activity, sex, length of intervention, and physical activity changes. Sensitivity analyses by removing each individual study from the meta-analysis were conducted to determine whether any particular study would significantly change the outcome estimates. Publication bias was evaluated using the Begg test and the Egger test at the *P* < .10 level of significance. If publication bias was detected, the trim-and-fill computation was used to assess the effect of publication bias on the interpretation of the outcome estimates.^34^ Unless otherwise specified, a *P* < 0.05 was considered significant. Statistical analyses were performed using STATA software (version 12.0; College Station, Texas, USA) and Review manager (version 5.2; the Nordic Cochrane Centre, Copenhagen, Denmark).

## RESULTS

### Study Characteristics

**FIGURE 1:**
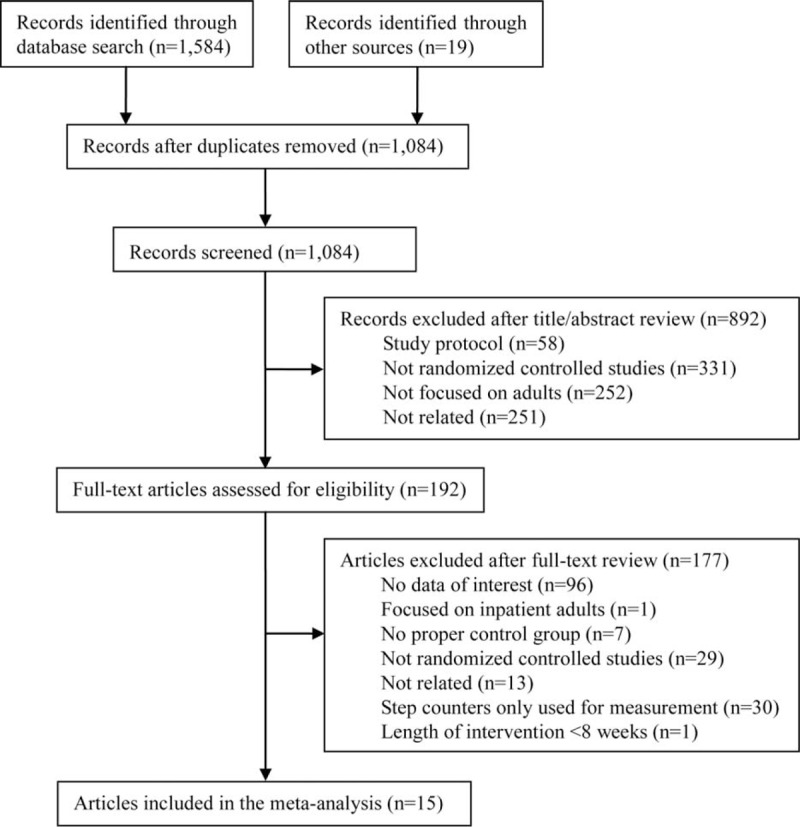
Flow diagram of literature search and study selection.

The literature search results and the study selection process are shown in Figure 1. In total, 1603 articles were identified, where 344 were from PubMed, 829 from Web of Science, 411 from the Cochrane Central Register of Controlled Trials, and 19 from identified systematic reviews or meta-analyses. After duplicate exclusion, title/abstract review, and full-text assessment, 15 studies met the inclusion criteria and were included in the final meta-analysis.^10–24^

The characteristics of the included studies are summarized in Table 1 . A total sample size of 3262 participants (1649 as intervention participants and 1613 as controls) was pooled from the 15 studies. Most of the included participants were overweight or obese, and physically inactive or sedentary. The length of step counter intervention varied from 8 to 48 weeks. All studies reported data on sedentary time, with 8 of them using subjective methods (6 with IPAQ and 2 with other questionnaires),^10,12–14,16,17,22,24^ 6 using objective methods (3 with accelerometer [details not provided], 2 with ActivPAL [PAL Technologies, Glasgow, Scotland], 1 with StepWatch [Orthocare Innovations, Mountlake Terrace, WA]),^11,15,19–21,23^ and 1 using both (IPAQ and accelerometer [details not provided]).^18^ Five studies clearly indicated that the step counter interventions were conducted in workplace settings.^11,14,17,19,24^ Seven studies reported adherence to step counter use among participants who completed the intervention, with an average rate of 79%. Eight studies had dropout rates <20%, while they were higher than 20% among the other studies except one that did not report.^24^ Three studies were from the United States,^11,15,19^ 9 from Europe,^10,14,17,18,20–24^ and 3 from Australia.^12,13,16^

**TABLE 1:**
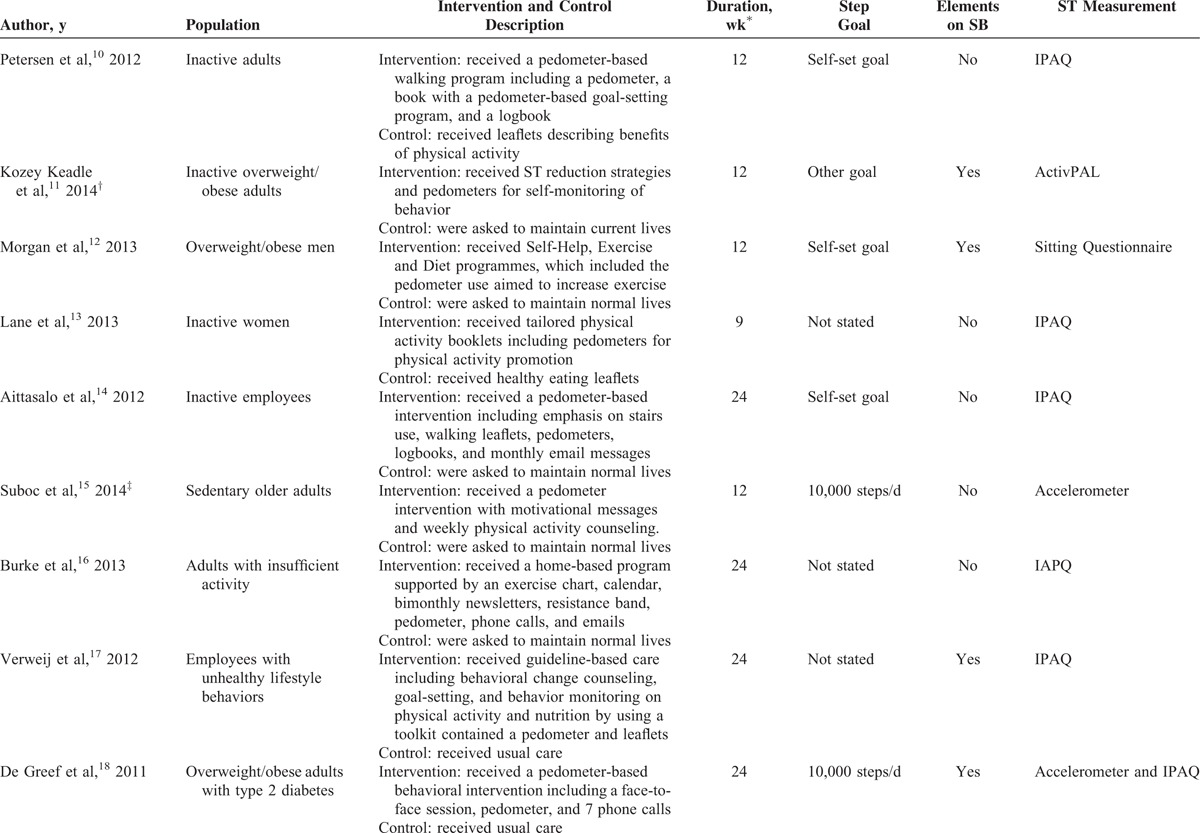
Characteristics of Each RCT

**TABLE 1 (Continued):**
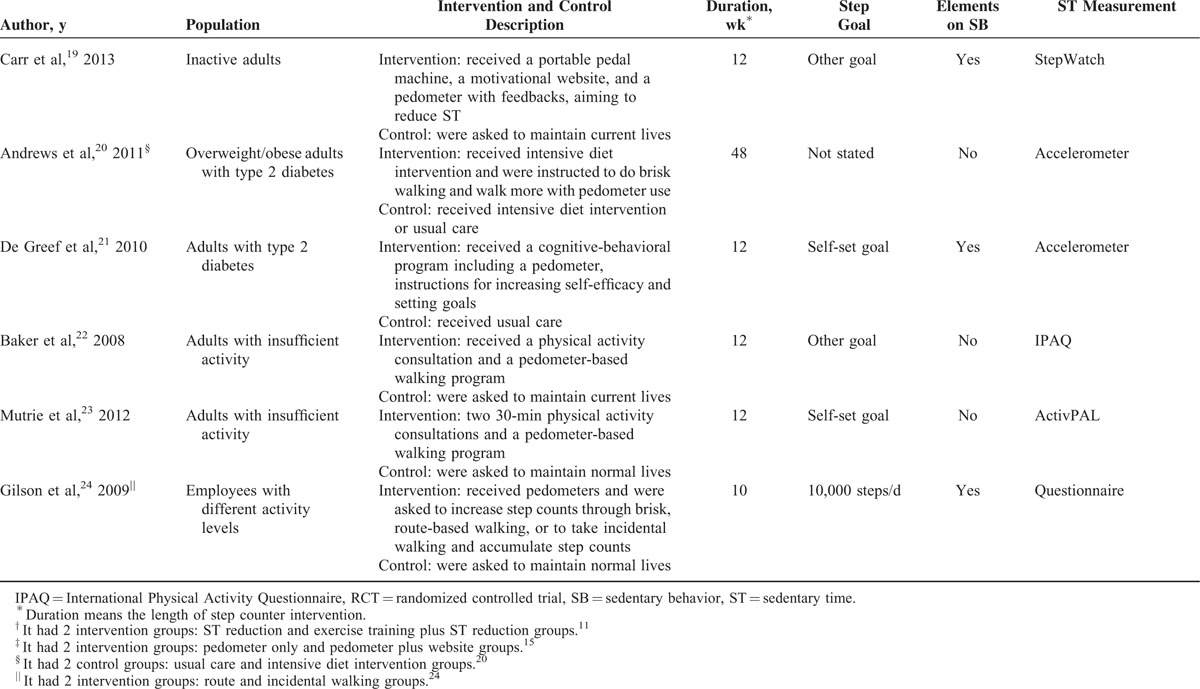
Characteristics of Each RCT

The risk-of-bias assessment for each study is described in Supplemental Digital Content 4, http://links.lww.com/MD/A390. The largest risk of bias came from attrition bias, with 7 of the included studies using improper methods in dealing with incomplete outcome data.^11,13–17,19^ Because of the nature of step counter intervention, which requires unsealed step counters to promote physical activity and to reduce sedentary time, complete blinding of participants and personnel is impossible and unnecessary, and therefore, the risk of performance bias was judged to be low.

### Meta-Analysis of Step Counter Use and Sedentary Time

**FIGURE 2:**
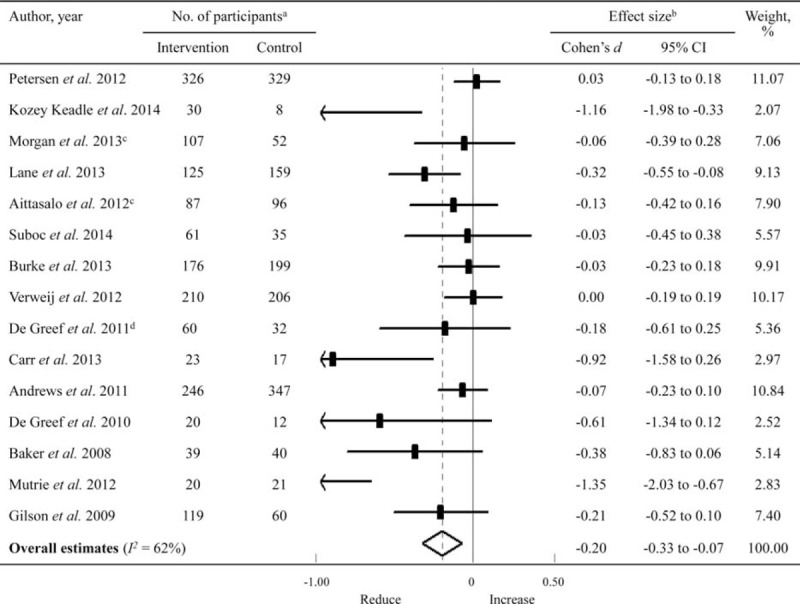
Forest plot examining the association between step counter use and sedentary time among adults. (A) The sample size represented the number of participants included in the per-protocol or intention-to-treat analyses. (B) Effect size was calculated using a random-effects model. (C) Data of sedentary time were imputed using the combined mean values of sedentary time on work and nonwork days. (D) Accelerometer-measured sedentary time was used in this meta-analysis. CI = confidence interval.

Fifteen studies involving a total of 3262 adults were pooled in this meta-analysis. Step counter use was associated with a small but significant overall reduction in sedentary time (*d* = −0.20, 95% CI −0.33 to −0.07; Figure 2), where the effect size was equaled to a reduction in sedentary time of ∼23 min/d compared with the controls. The Cochrane *Q* test indicated substantial heterogeneity among study results (*P* = 0.001), with an *I*^*2*^ value estimating that 62% of the variance is caused by between study differences.

Subgroup analyses showed that step counter use together with a step goal led to a significant reduction in sedentary time (*d* = −0.32, 95% CI −0.53 to −0.11), even though heterogeneity between studies remained high (*I*^*2*^ = 68%, *P* = 0.001); whereas without, there was only a trend toward reduced sedentary time (*d* = −0.09, 95% CI −0.21 to 0.04; *I*^*2*^ = 38.7%, *P* for heterogeneity 0.18) (Table 2). The overall effect sizes did not differ significantly (*P* = 0.42) between step counter use with (*d* = −0.29, 95% CI −0.54 to −0.04) or without (*d* = −0.17, 95% CI −0.33 to −0.01) interventions aiming to promote sedentary behavior changes (Table 2). Notably, studies using objective methods for sedentary time measurement showed a greater reduction in sedentary time compared with those using subjective methods (*d* = −0.52, 95% CI −0.88 to −0.15 vs *d* = −0.09, 95% CI −0.19 to 0.11, *P* = 0.03 for between-group comparison), whereas heterogeneity of the latter subgroup was low (*I*^*2*^ = 24.5%, *P* = 0.23).

**TABLE 2:**
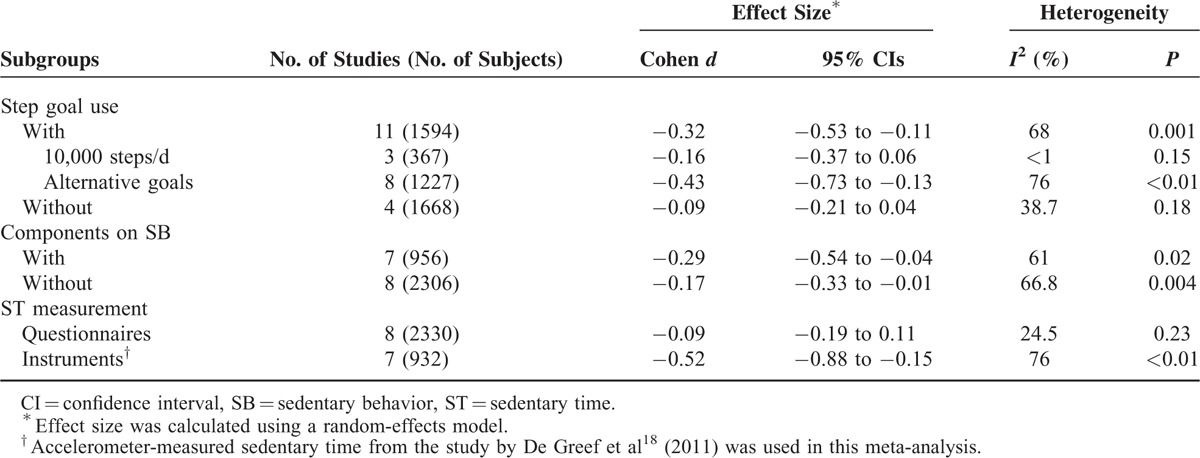
Subgroup Analyses

The random-effects meta-regression analyses showed that none of the following covariates was the potential predictor of changes in sedentary time: baseline age (β coefficient, −0.055, *P* = 0.93), baseline BMI (−0.039, *P* = 0.33), sex (−0.15, *P* = 0.66), length of intervention (0.009, *P* = 0.26), baseline sedentary time (−0.00003, *P* = 0.91), baseline walking steps (0.00004, *P* = 0.79), and changes in walking steps (−0.0001, *P* = 0.69). Besides, neither the baseline values nor change scores of light, moderate, vigorous, moderate to vigorous, or total physical activity were found to be associated with changes in sedentary time (Table 3). When studies were individually removed, the overall effect sizes remained largely unchanged. Statistical evidence of publication bias was found among these studies (Begg test, *P* = 0.001; Egger test, *P* = 0.01). However, the application of the trim and fill method did not identify any missing study (Figure, Supplemental Digital Content 5, http://links.lww.com/MD/A390, which shows the graph of the filled funnel plot) or change the overall effect size (*d* = −0.20, 95% CI −0.33 to −0.07).

**TABLE 3:**
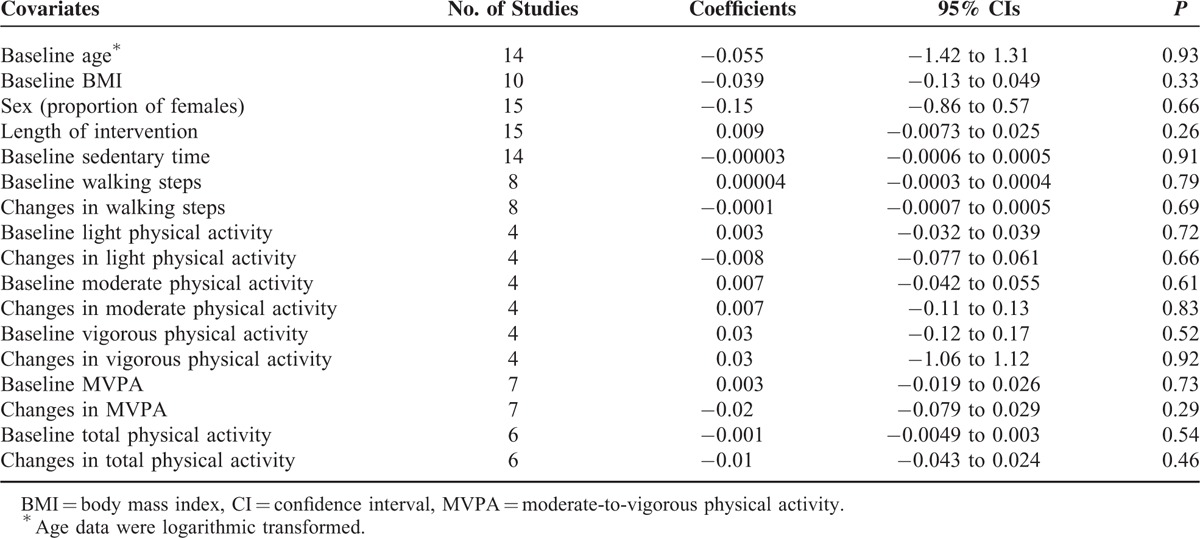
Univariate Weighted Random-Effects Meta-Regression Models

## DISCUSSION

This meta-analysis shows that among adults, step counter use was associated with a small but significant overall effect in reducing sedentary time compared with controls. It further shows that step goal setting was a key predictor of reduced sedentary time, whereas having interventions aiming to promote sedentary behavior changes seemed to be not. Furthermore, this study also shows that using objective methods (eg, accelerometers) for sedentary time measurement obtained a greater reduction in sedentary time compared with using subjective methods (eg, questionnaires), indicating that objective methods might have a higher sensitivity or accuracy than subjective methods in determining sedentary time.

Step counters are commonly used in walking programmes, and our results are partly in agreement with the findings from Prince et al,^35^ who showed that physical activity interventions were associated with a significant but small reduction in sedentary time among adults. However, the authors failed to specify the applied physical activity interventions. Among the characteristics of step counter interventions, having a step goal was found to be essential in reducing sedentary time, whereas the respective benefits of using different step goals in reducing sedentary time remain unclear. Interestingly, on the one hand, in studies where step counter users had a 10,000 steps/d goal, there was only an overall trend toward reduced sedentary time, which could also be seen in the individual studies. On the other hand, setting an alternative personalized step goal yielded significantly reduced sedentary time among step counter users, although the step goals varied substantially from each other. Therefore, to specify the goal use among step counter users is required in future studies.

It has been well documented that interventions with focus on reducing sedentary behaviors are associated with clinically meaningful reductions in sedentary time.^35^ This is reasonably in accordance with our findings that step counter use with interventions aiming to promote changes in sedentary behaviors was correlated with reduced sedentary time. However, it is worth noting that this recommendation might be unnecessary because a comparable reduction in sedentary time was observed in step counter users regardless of having such interventions or not. Yet it should be acknowledged that conclusions from indirect comparisons (subgroup analyses) are less reliable compared with those from head-to-head trials. Future studies using randomized controlled designs are worth being conducted to assess whether the supplementation of components on reducing sedentary behaviors to step counter use would give any additional benefits on sedentary time reduction.

The method of sedentary time measurement makes a substantial difference in the observed effect sizes in reducing sedentary time among step counter users, which may in part reflect a greater sensitivity or accuracy of objective methods in assessing sedentary time compared with subjective methods. However, it cannot be completely ruled out that this discrepancy might be also due to the potential differences of the step counter interventions (clinical heterogeneity). Subjective measurement using questionnaires such as IPAQ have been validated in epidemiological studies,^36^ but uncertainty remains regarding their relation to the objective measurement using devices such as accelerometers.^37–39^ Despite a moderate correlation observed between these 2 methods in measuring sedentary time, it is noted that the sedentary time is still largely underestimated using subjective versus objective methods.^38^ Besides, it should be noted that any of the objective methods that were used in the included studies of this meta-analysis cannot detect specific domains of sedentary time, such as the time spent using computers, watching TV, or sitting at work. Therefore, as also suggested by Healy et al,^40^ future studies would benefit from the incorporation of both methods, not only for measuring the total sedentary time, but also for capturing the domain-specific sedentary time and for exploring the patterns of sedentary time accumulation.

In recent years, there is an ongoing controversy with regard to the association between physical activity and sedentary time. The systematic review from Mansoubi et al suggested that the time from sedentary behaviors and light physical activity would be reallocated from one to another,^27^ which is sometimes also referred to “displacement hypothesis.” However, Pearson et al^41^ pointed it out in a more conservative way that, although sedentary behaviors are inversely associated with physical activity, their relationship is so weak that the reallocation of time from sedentary behaviors to physical activity should not be considered in a simplified or direct manner. Besides, a cross-sectional survey has shown that the patterns in physical activity and sedentary behaviors may be mutually exclusive, indicating that there might be no real correlation between the time spent being physically active or sedentary.^8^ Partly in line with these, our meta-regression analyses also do not show any clear or strong evidence regarding the association between changes in sedentary time and any forms of physical activity.

The current findings from our study provide evidence in support of the widespread recommendation and adoption of step counter use in health promotion programmes.^42^ Moreover, our study gives a potential explanation for the observed health benefits resulting from step counter use including weight loss^26,43^ because epidemiological evidence suggests that decreased sedentary time is associated with reduced BMI,^44^ in which reduction is assumed to be independent of increased physical activity.^43^ In addition, there is emerging evidence that frequent interruptions from sedentary time are associated with an improved metabolic profile including reduced waist circumference and BMI.^45^ It seems likely that weight loss resulted from step counter use could be also related to the breaks in sedentary time, at least partly. However, to date, very few studies with very small sample sizes have focused on this topic and showed only some trends.^9,11^ More RCTs with larger sample sizes are therefore required to address this concern in future.

The main strengths of this meta-analysis include an extensive literature search, a reasonably large sample size, and comprehensive summaries of the effects of step counter use on sedentary time, along with the exploration of heterogeneity using a broad range of study and intervention characteristics as covariates. However, when interpreting these results, several limitations must be considered. First, the search strategy used in this meta-analysis was restricted to English-language studies, which might lead to a language or cultural bias. Moreover, a publication bias was detected using either Begg test or Egger test, increasing the risk of reporting bias resulting from possible small-study effects.^31^ However, the trim and fill method suggested that, the overall effect size derived from the currently included studies was unlikely to be affected by publication bias. Second, as indicated in the previous studies^25,26^ because step counter interventions generally included some other components (eg, step goals, promotions of sedentary behavior changes), it is difficult to establish their independent contributions to the observed effect sizes. Third, there existed some evidence of heterogeneity across studies, which were just partly explained by the use of step goals and the methods used for sedentary time measurement (subgroup analyses). This would somehow weaken the robustness of our main findings. Moreover, meta-regression analyses might have limited power to detect significant predictors that moderate the sedentary time changes related to pedometer intervention or explain the potential sources of heterogeneity.^31^ Fourth, the high dropout rates and the attrition bias reported in this meta-analysis might downgrade the evidence level. Fifth, although this meta-analysis shows that step counter use was associated with an overall reduction in sedentary time of ∼23 min/d, it remains unknown whether changes of this magnitude are sufficient to benefit healthy outcomes. Finally, this meta-analysis fails to show evidence regarding the effects of step counter use in workplace settings in reducing sedentary time among adults because several included studies did not clearly state whether the step counters were used for workplace interventions.^15,18,22^

In summary, this study provided evidence that step counter use leads to reduced sedentary time in adults, and the step goal setting is an important predictor of reduced sedentary time. Future studies are required to specify the goal use among step counter users to employ objective and subjective methods for measuring both total and domain-specific sedentary time, to address the magnitude of reductions in sedentary time sufficient to benefit healthy outcomes, and to investigate the effects of step counter use on breaking up the prolonged sedentary time to gain additional health benefits.

## References

[1] Sedentary Behaviour Research N. Letter to the editor: standardized use of the terms “sedentary” and “sedentary behaviours”. *Appl Physiol Nutr Metab* 2012; 37:540–542. 22540258 10.1139/h2012-024

[2] Hancox RJ Milne BJ Poulton R Association between child and adolescent television viewing and adult health: a longitudinal birth cohort study. *Lancet* 2004; 364:257–262. 15262103 10.1016/S0140-6736(04)16675-0

[3] Scheers T Philippaerts R Lefevre J SenseWear-determined physical activity and sedentary behavior and metabolic syndrome. *Med Sci Sports Exerc* 2013; 45:481–489. 23034646 10.1249/MSS.0b013e31827563ba

[4] Wilmot EG Edwardson CL Achana FA Sedentary time in adults and the association with diabetes, cardiovascular disease and death: systematic review and meta-analysis. *Diabetologia* 2012; 55:2895–2905. 22890825 10.1007/s00125-012-2677-z

[5] Boyle T Fritschi L Heyworth J Long-term sedentary work and the risk of subsite-specific colorectal cancer. *Am J Epidemiol* 2011; 173:1183–1191. 21421743 10.1093/aje/kwq513

[6] Biswas A Oh PI Faulkner GE Sedentary time and its association with risk for disease incidence, mortality, and hospitalization in adults: a systematic review and meta-analysis. *Ann Intern Med* 2015; 162:123–132. 25599350 10.7326/M14-1651

[7] Colley RC Garriguet D Janssen I Physical activity of Canadian adults: accelerometer results from the 2007 to 2009 Canadian Health Measures Survey. *Health Rep* 2011; 22:7–14. 21510585

[8] Craft LL Zderic TW Gapstur SM Evidence that women meeting physical activity guidelines do not sit less: an observational inclinometry study. *Int J Behav Nutr Phys Act* 2012; 9:122. 23034100 10.1186/1479-5868-9-122 PMC3490758

[9] Parry S Straker L Gilson ND Participatory workplace interventions can reduce sedentary time for office workers—a randomised controlled trial. *PLoS One* 2013; 8:e78957. 24265734 10.1371/journal.pone.0078957 PMC3827087

[10] Petersen CB Severin M Hansen AW A population-based randomized controlled trial of the effect of combining a pedometer with an intervention toolkit on physical activity among individuals with low levels of physical activity or fitness. *Prev Med* 2012; 54:125–130. 22200586 10.1016/j.ypmed.2011.12.012

[11] Kozey Keadle S Lyden K Staudenmayer J The independent and combined effects of exercise training and reducing sedentary behavior on cardiometabolic risk factors. *Appl Physiol Nutr Metab* 2014; 39:770–780. 24971677 10.1139/apnm-2013-0379 PMC4075057

[12] Morgan PJ Callister R Collins CE The SHED-IT community trial: a randomized controlled trial of internet- and paper-based weight loss programs tailored for overweight and obese men. *Ann Behav Med* 2013; 45:139–152. 23129021 10.1007/s12160-012-9424-z

[13] Lane A Murphy N Bauman A An effort to “leverage” the effect of participation in a mass event on physical activity. *Health Promot Int* 2013. 10.1093/heapro/dat077 24226297

[14] Aittasalo M Rinne M Pasanen M Promoting walking among office employees—evaluation of a randomized controlled intervention with pedometers and e-mail messages. *BMC Public Health* 2012; 12:403. 22672576 10.1186/1471-2458-12-403 PMC3444317

[15] Suboc TB Strath SJ Dharmashankar K Relative importance of step count, intensity, and duration on physical activity's impact on vascular structure and function in previously sedentary older adults. *J Am Heart Assoc* 2014; 3:e000702. 24572255 10.1161/JAHA.113.000702 PMC3959701

[16] Burke L Lee AH Jancey J Physical activity and nutrition behavioural outcomes of a home-based intervention program for seniors: a randomized controlled trial. *Int J Behav Nutr Phys Act* 2013; 10:14. 23363616 10.1186/1479-5868-10-14 PMC3568722

[17] Verweij LM Proper KI Weel ANH The application of an occupational health guideline reduces sedentary behaviour and increases fruit intake at work: results from an RCT. *Occup Environ Med* 2012; 69:500–507. 22383591 10.1136/oemed-2011-100377

[18] De Greef KP Deforche BI Ruige JB The effects of a pedometer-based behavioral modification program with telephone support on physical activity and sedentary behavior in type 2 diabetes patients. *Patient Educ Couns* 2011; 84:275–279. 20732776 10.1016/j.pec.2010.07.010

[19] Carr LJ Karvinen K Peavler M Multicomponent intervention to reduce daily sedentary time: a randomised controlled trial. *BMJ Open* 2013 e003261. 10.1136/bmjopen-2013-003261 PMC3808782 24141969

[20] Andrews RC Cooper AR Montgomery AA Diet or diet plus physical activity versus usual care in patients with newly diagnosed type 2 diabetes: the Early ACTID randomised controlled trial. *Lancet* 2011; 378:129–139. 21705068 10.1016/S0140-6736(11)60442-X

[21] De Greef K Deforche B Tudor-Locke C A cognitive-behavioural pedometer-based group intervention on physical activity and sedentary behaviour in individuals with type 2 diabetes. *Health Educ Res* 2010; 25:724–736. 20338978 10.1093/her/cyq017 PMC2936553

[22] Baker G Gray SR Wright A The effect of a pedometer-based community walking intervention “Walking for Wellbeing in the West” on physical activity levels and health outcomes: a 12-week randomized controlled trial. *Int J Behav Nutr Phys Act* 2008; 5:44. 18775062 10.1186/1479-5868-5-44 PMC2546435

[23] Mutrie N Doolin O Fitzsimons CF Increasing older adults’ walking through primary care: results of a pilot randomized controlled trial. *Fam Pract* 2012; 29:633–642. 22843637 10.1093/fampra/cms038 PMC3501246

[24] Gilson ND Puig-Ribera A McKenna J Do walking strategies to increase physical activity reduce reported sitting in workplaces: a randomized control trial. *Int J Behav Nutr Phys Act* 2009; 6:43. 19619295 10.1186/1479-5868-6-43 PMC2717045

[25] Qiu S Cai X Chen X Step counter use in type 2 diabetes: a meta-analysis of randomized controlled trials. *BMC Med* 2014; 12:36. 24571580 10.1186/1741-7015-12-36 PMC4016223

[26] Bravata DM Smith-Spangler C Sundaram V Using pedometers to increase physical activity and improve health: a systematic review. *JAMA* 2007; 298:2296–2304. 18029834 10.1001/jama.298.19.2296

[27] Mansoubi M Pearson N Biddle SJH The relationship between sedentary behaviour and physical activity in adults: a systematic review. *Prev Med* 2014; 69:28–35. 25193005 10.1016/j.ypmed.2014.08.028

[28] Moher D Liberati A Tetzlaff J Preferred Reporting Items for Systematic Reviews and Meta-Analyses: the PRISMA statement. *PLoS Med* 2009; 6:e1000097. 19621072 10.1371/journal.pmed.1000097 PMC2707599

[29] Qiu S Cai X Schumann U Impact of walking on glycemic control and other cardiovascular risk factors in type 2 diabetes: a meta-analysis. *PLoS One* 2014; 9:e109767. 25329391 10.1371/journal.pone.0109767 PMC4201471

[30] Chudyk A Petrella RJ Effects of exercise on cardiovascular risk factors in type 2 diabetes: a meta-analysis. *Diabetes Care* 2011; 34:1228–1237. 21525503 10.2337/dc10-1881 PMC3114506

[31] Higgins JPT Green S (Eds): *Cochrane Handbook for Systematic Reviews of Interventions version 5.1.0*. The Cochrane Collaboration. http://handbook.cochrane.org/ Updated March 2011. Accessed 17 July 2015.

[32] Hozo SP Djulbegovic B Hozo I Estimating the mean and variance from the median, range, and the size of a sample. *BMC Med Res Methodol* 2005; 5:13. 15840177 10.1186/1471-2288-5-13 PMC1097734

[33] Cohen J Statistical Power Analyses for the Behavioral Sciences. 2nd ed. New York, NY: Erlbaum; 1988.

[34] Duval S Tweedie R Trim and fill: A simple funnel-plot-based method of testing and adjusting for publication bias in meta-analysis. *Biometrics* 2000; 56:455–463. 10877304 10.1111/j.0006-341x.2000.00455.x

[35] Prince SA Saunders TJ Gresty K A comparison of the effectiveness of physical activity and sedentary behaviour interventions in reducing sedentary time in adults: a systematic review and meta-analysis of controlled trials. *Obes Rev* 2014; 15:905–919. 25112481 10.1111/obr.12215 PMC4233995

[36] Craig CL Marshall AL Sjostrom M International Physical Activity Questionnaire: 12-country reliability and validity. *Med Sci Sports Exerc* 2003; 35:1381–1395. 12900694 10.1249/01.MSS.0000078924.61453.FB

[37] Atkin AJ Gorely T Clemes SA Methods of measurement in epidemiology: sedentary behaviour. *Int J Epidemiol* 2012; 41:1460–1471. 23045206 10.1093/ije/dys118 PMC3465769

[38] Dyrstad SM Hansen BH Holme IM Comparison of self-reported versus accelerometer-measured physical activity. *Med Sci Sports Exerc* 2014; 46:99–106. 23793232 10.1249/MSS.0b013e3182a0595f

[39] Boyle T Lynch BM Courneya KS Agreement between accelerometer-assessed and self-reported physical activity and sedentary time in colon cancer survivors. *Support Care Cancer* 2015; 23:1121–1126. 25301224 10.1007/s00520-014-2453-3

[40] Healy GN Clark BK Winkler EA Measurement of adults’ sedentary time in population-based studies. *Am J Prev Med* 2011; 41:216–227. 21767730 10.1016/j.amepre.2011.05.005 PMC3179387

[41] Pearson N Braithwaite RE Biddle SJH Associations between sedentary behaviour and physical activity in children and adolescents: a meta-analysis. *Obes Rev* 2014; 15:666–675. 24844784 10.1111/obr.12188 PMC4282352

[42] Colberg SR Albright AL Blissmer BJ Exercise and type 2 diabetes: American College of Sports Medicine and the American Diabetes Association: joint position statement. *Diabetes Care* 2010; 33:e147–e167. 21115758 10.2337/dc10-9990 PMC2992225

[43] Richardson CR Newton TL Abraham JJ A meta-analysis of pedometer-based walking interventions and weight loss. *Ann Fam Med* 2008; 6:69–77. 18195317 10.1370/afm.761 PMC2203404

[44] Stamatakis E Hamer M Tilling K Sedentary time in relation to cardio-metabolic risk factors: differential associations for self-report vs accelerometry in working age adults. *Int J Epidemiol* 2012; 41:1328–1337. 22634868 10.1093/ije/dys077

[45] Healy GN Dunstan DW Salmon J Breaks in sedentary time: beneficial associations with metabolic risk. *Diabetes Care* 2008; 31:661–666. 18252901 10.2337/dc07-2046

